# Simultaneous editing of TCR, HLA-I/II and HLA-E resulted in enhanced universal CAR-T resistance to allo-rejection

**DOI:** 10.3389/fimmu.2022.1052717

**Published:** 2022-12-02

**Authors:** Wuling Li, Xiuxiu Zhu, Yanmin Xu, Jun Chen, Hongtao Zhang, Zhi Yang, Yanan Qi, Juan Hong, Yunyan Li, Guixue Wang, Junjie Shen, Cheng Qian

**Affiliations:** ^1^ Key Laboratory for Biorheological Science and Technology of Ministry of Education, College of Bioengineering, Chongqing University, Chongqing, China; ^2^ Center for Precision Medicine of Cancer, Chongqing Key Laboratory of Translational Research for Cancer Metastasis and Individualized Treatment, Chongqing University Cancer Hospital, Chongqing, China; ^3^ Chongqing Key Laboratory of Gene and Cell Therapy, Institute of Precision Medicine and Biotechnology, Chongqing Precision Biotech Co., Ltd., Chongqing, China; ^4^ Key Laboratory for Biorheological Science and Technology of Ministry of Education, State and Local Joint Engineering Laboratory for Vascular Implants, Bioengineering College of Chongqing University, Chongqing, China

**Keywords:** universal CAR, CRISPR/Cas9, ETUCAR-T, natural killer cell, HLA-E

## Abstract

**Introduction:**

The major challenge for universal chimeric antigen receptor T cell (UCAR-T) therapy is the inability to persist for a long time in patients leading to inferior efficacy clinically. The objective of this study was to design a novel UCAR-T cell that could avoid the occurrence of allo-rejection and provide effective resistance to allogeneic Natural Killer (NK) cell rejection, together with the validation of its safety and efficacy *ex vivo* and *in vivo*.

**Methods:**

We prepared T-cell receptor (TCR), Human leukocyte antigen (HLA)-I/II triple-edited (TUCAR-T) cells and evaluated the anti-tumor efficacy ex vivo and in vivo. We measured the resistance of exogenous HLA-E expressing TUCAR-T (ETUCAR-T) to NK rejection by using an enhanced NK. Furthermore, we established the safety and efficacy of this regimen by treating Nalm6 tumor-bearing mice with a repeated high-dose infusion of ETUCAR-T. Moreover, we analyzed the effects of individual gene deficiency CAR-T on treated mice and the changes in the transcriptional profiles of different gene-edited T cells *via* RNA-Seq.

**Results:**

Data showed that HLA-II editing didn’t impair the anti-tumor efficacy of TUCAR-T ex vivo and in vivo and we found for the first time that HLA-II deficiency could facilitate the persistence of CAR-T. Contrastively, as the most commonly eliminated target in UCAR-T, TCR deficiency was found to be a key disadvantageous factor for the shorter-term anti-tumor efficacy *in vivo*. Our study demonstrated ETUCAR-T could effectively resist allogeneic NK rejection *ex vivo* and *in vivo*.

**Discussion:**

Our research provided a potential and effective strategy for promoting the persistence of UCAR-T cells in clinical application. And it reveals the potential key factors of the poor persistence of UCAR-T along with new insights for future development.

## Introduction

Revolutionary advances in cancer treatment by chimeric antigen receptor T-cell (CAR-T) therapy have been achieved, especially in hematological malignancies (1). Hundreds of preclinical and clinical trials on CAR-T therapy have been conducted worldwide. Up to now, six CAR-T products have been approved by the Food and Drug Administration (FDA) for the clinical treatment of hematological tumors. Nevertheless, this therapy has not been widely applied in cancer treatment due to the high cost and the long time consumption of individualized manufacturing in the production of autologous CAR-T cells. The development of off-the-shelf universal CAR-T (UCAR-T) therapy is considered as an attractive direction. However, UCAR-T therapy also faces the challenges of uncertain gene-editing operation regimes and a wide gap in clinical efficacy compared to traditional unedited autologous CAR-T.

By now, the reported strategies for UCAR-T therapy are based on the combination of knocking out the T-cell receptor (TCR) and clearing lymphocytes by the CD52 monoclonal antibody or simultaneously eliminating β2 microglobulin (B2M) and/or programmed cell death protein 1 (PD-1) by means of zinc finger nucleases (ZFNs) and transcription activator-like effector nucleases (TALENs) as well as clustered regularly interspaced short palindromic repeats/Cas9 protein (CRISPR/Cas9) (2). CRISPR/Cas9 is considered as a more favorable selection because of the superiority in single-target and higher editing efficiency. Since the first clinical study on UCAR-T therapy, which started in 2015, reported the achievement of molecular remission within 28 days in two cases of infantile leukemia (3), increasing clinical studies focusing on UCAR-T therapy have ensued (4, 5).

HLA-II molecules, which are mainly expressed on the surface of antigen-presenting cells (APCs), play an important role in organ transplantation. In the field of UCAR, Kagoya et al. found that HLA-II expression on activated T cells would rise to a varying level, by up to 50% (6). Importantly, our prior clinical studies also found that up to 90% of HLA-II was detected in ready-to-infuse autologous CAR-T. The inconsistent HLA-II^pos^ may be attributed to the different activation and stimulation approach during T-cell production. However, both findings support the necessity of HLA-II elimination (6). We then restrained the expression of HLA-II by editing the class II transactivator (CIITA) with CRISPR/Cas9 (7) and successfully obtained TCR/HLA-I/HLA-II triple-deficiency UCAR-T (TUCAR-T) cells. Interestingly, HLA-II deficiency was found to improve rather than attenuate the efficacy of CAR-T cells. Furthermore, we exogenously delivered an HLA-E gene, a member of HLA-I family, and generated ETUCAR-T that could escape from the attack of host NK cells (8, 9). The safety and efficacy of ETUCAR-T cells were fully tested both *ex vivo* and *in vivo*, and the results of multiple dosing in mice have been provisionally provided as a reference for clinical application.

Despite the improvements in several aspects, our data suggested that ETUCAR-T showed unsatisfactory persistence in NOD.CgPrkdcscidIl2rgtm1Sug/JicCrl (NOG) mice. We thus further investigated and obtained some novel insights about the differential impacts of the deficiencies of TCR, HLA-I, and HLA-II on CAR-T cells by whole transcriptional profiling using RNA-seq. To sum up, discoveries in our research provided significant evidence for revealing the key factors affecting UCAR-T function and provided us with new countermeasures for UCAR-T therapy in the future.

## Materials and methods

### Cells and culture conditions

PBMCs were isolated from healthy volunteer donors using a human peripheral blood lymphocyte separation solution (TBDscience Tianjin, China). Primary human T cells were isolated by the Pan T Cell Isolation Kit, human (Miltenyi Biotech, Bergisch Gladbach) and stimulated with Dynabeads™ CD3/CD28 (Invitrogen, USA) at a density of 2 × 10^6^ cells/ml in an immunocell medium (TBDscience Tianjin, China) with 10% fetal bovine serum (FBS) (Biological Industries Beit-Haemek, Israel), 50 IU/ml IL7, 50 IU/ml IL15, and 50 IU/ml IL21 (Peprotech, USA). Dynabeads were removed with a magnetic holder at 2~3 days after activation. CAR-T cells were cryopreserved at day 9 postactivation in a lab-created cryoprotectant for injection at 1 × 10^8^ cells per vial. NK cells were isolated from PBMCs using human CD56 MicroBeads (Miltenyi Biotech, Bergisch Gladbach) and LS Columns (Miltenyi Biotech, Bergisch Gladbach) by the manufacturer’s instructions (Miltenyi Biotech, Bergisch Gladbach) and cultured at a density of 1 × 10^6^ cells/ml in an immunocell medium (TBDscience Tianjin, China) supplemented with 10% FBS (Biological Industries, Israel), 50I U/ml IL18 and 50 IU/ml IL2 (Peprotech, USA). NK was transduced with a lentiviral expression of membrane-bound IL15 at 2 ~ 3 days of activation, and experiments were performed at 9 days of NK activation.

All cell lines were STR-fingerprinted and validated to be mycoplasma-free by PCR. The human acute lymphoblastic leukemia cell line CD19^+^ Nalm6, human chronic myeloid leukemia cell line CD19^-^ K562, and human carcinoma cell line A549 were purchased from ATCC (Virginia, USA). The A549 cell line was transduced with CD19 antigen in the Pcdh vector using the lentiviral vector to create a new CD19^+^ A549 cell line. Nalm6 and K562 cell lines were transduced with Luc-2A-GFP in the Pcdh vector using the lentiviral to create the new cell line Nalm6-Luc-GFP. Nalm6 and K562 were cultured in RPMI 1640 (Gibco, USA), and 293T and A549 were cultured in DMEM (Gibco, USA). All cell lines were cultured with a medium supplemented with 10% FBS and 100 IU/ml penicillin/streptomycin (Beyotime Shanghai, China).

### Generation of constructs

CD19 CAR was synthesized and/or amplified by PCR as published based on sequencing information and subcloned into a lentiviral vector (10). Mutant HLA-E was a fusion protein consisting of a codon-optimized signal peptide of β2-microglobulin (Genscript, Nanjing, China) and HLA-E Cdna Open Reading Frame (ORF) Clone in Cloning Vector, Human (Sinobiological, China). The following primers were used in overlap PCR: β2-microglobulin forward (5′-GCTCTAGAATGAGCAGAAGCGT-3′) and reverse (5′-TACTTCAAGGAGTGGGAGCCCATGCTAGGA ATTCGCTTCC-3′), HLA-E Cdna ORF forward (5′-GGCTCCCACTCCTT GAAGTATTTCCACACTTCCGTGTCCC-3′) and reverse (5′-GGGTGTACATTACAAGCTGT-3′).

### Flow cytometry

Flow cytometry (FCM) results were acquired on a LSRFortessa™ (Becton, Dickinson and Company, USA) or Quanteon (Agilent, USA) and analyzed by FlowJo_v10.6.2 or NovoExpress 1.4.1. Non-transduced T cells (Ctrl-T) and isotype antibodies were used as controls. The Human Leukocyte Antigen (HLA)-DR antibody is used to detect HLA-II expression levels on the cell surface, and the β2-microglobulin or HLA class I antibody is used to detect HLA-I expression levels on the cell surface. Information on the antibodies used in this study is shown in the **Supplementary Material**. CD3, HLA-I, and HLA-DR triple-negative UCAR-T cells were isolated by the flow cytometry instrument FACSAria III (BD) on day 7 postactivation.

### Clustered regularly interspaced short palindromic repeats design

The following genome targeting sequences were used in the study: TRAC: 5′-AGAGTCTCTCAGCTGGTACA-3′, B2M: 5′-GGCCGAGATGTCTCGCTCCG-3′, CIITA: 5′-GATATTGGCATAAGCCTCCC-3′. Primary human T cells were transduced with the CD19 CAR lentivirus at 24 h of activation and electroporated using 4D-NucleofectorTM X (Lonza, Germany) with RNP that was separately mixed by Cas9 protein (Gibco, USA) and chemically synthesized no-annealing-needed sgRNA (Genscript, China, bearing 2′-O-methyl at three first and last bases, 3′ phosphorothioate-modified bounds between three first and last bases) at a 1:1:1:3 mole ratio for 10–15 min at room temperature at 48 h postactivation.

### Lentivirus production

Lentiviruses were collected from the supernatants of 293T cells transduced with the lentivirus vector and helper plasmids (PMD2.G, pMDLg/Prre, and Prsv-Rev) as we described previously (11). After harvesting the supernatant, the lentivirus was mixed with 50% Polyethylene Glycol (PEG) and 4M NaCl at a 6:2:1 ratio and centrifuged at 10,000 × g at 4°C for 1 h. The supernatant was discarded following centrifugation, and the precipitate was dissolved in an appropriate volume of saline. For all experiments related to lentiviral transduction, the multiplicity of infection used was 2 MOI.

### On-target and predicted off-target Sanger sequencing

The genome of UCAR-T cells from three healthy donors was extracted, the on-targets or predicted off-targets fragments were amplified separately with their corresponding primers, and the fragments were ligated to the T vector (Takara, Japan) for sequencing. The on-target and predicted off-target primers for PCR amplified are listed in **Supplementary Experimental Methods**.

### Luciferase-based Cytotoxic T Lymphocyte (CTL) assay

In a 96-well, U-bottom plate (NEST, USA), CAR-T cells (effectors) and Nalm6-Luc-GFP (targets) or K562-Luc-GFP (targets) were cultured together at 37°C for 24 h at various effector- to-target ratios (E:T or E/T); the targets were 1 × 10^4^/well. Supernatants were harvested for cytokine secretion detection following the centrifugation of the plate. Avoiding the unequal transduction of CAR-positive in T cells, non-transduced Ctrl-T cells were supplemented to adjust both the number of CAR^+^ T cells, and the total number of T cells remained consistent in all groups. The substrate was added with the DPPIV-Glo™ Protease Assay (Promega, USA) and immediately centrifuged and detected. The results are reported as the percentage of killing based on the luciferase activity in the wells with tumor cells but without T cells [% killing=100−((RLU from well with effector and target cell coculture)/(RLU from well with target cells) ×100)].

### Real-time cell analysis CTL assay

A cytotoxicity assay to test CAR-T cells with adherent target cells was operated using an electrical impedance–based approach, namely, xCELLigence real-time cell analysis (RTCA) SP/MP Analyzer (Roche, Switzerland). The cell index represents the relative change of the cell proliferation rate for several days of continuous monitoring. Firstly, the baseline measurement was operated by adding 50 µl of DMEM per well to E-plates (Roche, Switzerland). Then, 100 μl of DMEM containing 1×10^4^ CD19^+/-^ A549 target cells were added in E-plates per well, and electrical impedance was measured throughout the cultivation period with 15 min intervals throughout the culture period until the target cells were in logarithmic growth (total time: 12 h). Next, CAR-T cells (effectors) were plated at a 1:1 E/T ratio in E-plates in a volume of 100 µl per well, following by discarding 50 µl of the medium. Negative control was described above.

### ELISA assays

The incubation supernatant was stored at -80°C. Samples were diluted in an appropriate ratio (the standard curve ranges from 30 to 300 pg/ml), and each sample was assayed in duplicate or triplicate using an IFN gamma Human Uncoated ELISA Kit (Invitrogen, USA). Data analysis was conducted according to the related protocol and algorithm by Varioskan LUX (Thermo Fisher Scientific). All data were within the range of the calibrated curves.

### Allogeneic rejection analyzed

Donor CAR-T cells were cocultured with freshly isolated allogeneic PBMCs at the specified E/T ratios in a 200 µl RPMI 1640 medium supplemented with 10% FBS in U-bottomed, 96-well plates. To generate primed alloreactive T cells in a host-versus-graft reaction (HvGR), donor CAR-T cells were treated with mitomycin C (BioVision, USA) in 10 μg/ml and then stained with the 2 mM CellTrace CFSE Cell Proliferation Kit (CFSE) (Thermo Fisher Scientific), mixed with fresh allogeneic PBMCs that were stained with a 2 mM CellTrace Violet Cell Proliferation Kit (CTV) (Thermo Fisher Scientific) at a 1:1 ratio. Cell stimulation was analyzed by FCM on day 0 and 7 days later. On the contrary, fresh allogeneic PBMCs were treated with mitomycin C (BioVision, USA) in 10 μg/ml mixed with donor CAR-T cells at a 1:1 ratio in an RPMI 1640 medium supplemented with 10% FBS in a graft-versus-host reaction (GvHR).

### Mouse xenograft studies

The Nalm6 tumor model established: 8~10-week-old NOG mice line NOD. Cg-PrkdcscidIl2rgtm1Sug/JicCrl (GemPharmatech, China) was transplanted intravenously with 5 × 10^5^ Nalm6-Luc-GFP tumor cells in the tail vein. CAR-T cells (2 × 10^6^, activation for 9 days) were infused 3 days later. Euthanasia was administered when necessary. In **Figures 3F, G** and **Figures S2E, F**, tumors were established in NOG mice (n = 3 per group) by the intravenous injection of 5 × 10^5^ Nalm6- Luc-GFP cells on day -3. Beginning on day 0, UCAR-T cells (2 × 10^6^) were infused with a single injection. Ctrl-T cells were injected as the control group. NK^mbIL15^ was injected 6 h before UCAR-T cell injection; the same volume of saline was injected into the T-cell-only infusion groups. The ratio of NK^mbIL15^: UCAR-T is 1:1 (by total cell count). All mice passed the qualifying quarantine a week before the experiment was conducted. To evaluate the development of xenogeneic graft-versus-host disease (GvHD), T-cell infused mice were monitored at least three times a week for clinical symptoms. In parallel, we have followed the proper previous reports of the performance of xenograft GvHD in mice (12).

### Real-time PCR

Blood samples or the spleen and bone marrow were obtained according to the trial procedure for CAR copy number detection. Genomic DNA was extracted from the samples using a QIAamp DNA Blood Mini Kit (Qiagen, Germany) and following the protocol as per instructions. We applied SYBR and TaqMan probes for qPCR in an ABI QuantStudio (Thermo Fisher Scientific). For CAR copy number detection, the TaqMan primers of forward 5′-CAGAAGAAGAAGAAGGAGGATGTG-3′ and reverse 5′- TACTCCTCTCTTCGTCCTAGATTG -3′ were used. The probe used was 5′-FAM- CTGAGAGTGAAGTTC-3′. The TaqMan method was performed in accordance with the published protocol (10). PCBP2 was used as a control, and a correction factor (CF) was generated to correct for the DNA copy number. DNA samples from healthy donors were detected as negative controls. A lower limit of quantification (LLOQ) of five copies per microliter of genomic DNA was determined.

Total RNA was extracted from cells using the RNeasy Mini Plus Kit (Qiagen, Germany) following the instructions and was reverse-transcribed to cDNA by PrimeScript RT reagents (TaKaRa, Japan). The following primers were used: NR4A3 forward 5′-GCAAGGGCTTTTTCAAGAGAACA‐3′ and reverse 5′-TTTGGAAGGCAGACGACCTC-3′, EGR3 forward 5′-TGCTATGACCGGCAAACTCG-3′ and reverse 5′-CCGATGTCCATTACATTCTCTGT-3′, CD70 forward 5′-GTCACTTGGGTGGGACGTAG-3′ and reverse 5′-GATGGATACGTAGCTGCCCC-3′, POLR2L forward 5′-TACGCTGCTTCACTTGTGGC-3′ and reverse 5′‐AGCGCATCCCCC TCGGT-3′, ID2 forward 5′-ATCCTGTCCTTGCAGGCTTC-3′ and reverse 5′-ACCGCTTATTCAGCCACACA-3′, FHL2 forward 5′-TCAGTGCAAAAAGCCCATCAC-3′ and reverse 5′-GCAGTAGGCAAAGTCATCGC-3′, HSPA5 forward 5′-GGACCACCTACTCCTGCGTC-3′ and reverse 5′-TCAAAGACCGTGTTCTCGGG-3′.

### RNA-seq

Total RNA was extracted from cells using the RNeasy Mini Plus Kit (Qiagen, Germany) on day 9 of activation, followed by fragmentation into small pieces with a fragment buffer at an appropriate temperature. The RNA library was constructed by the MGIEasy RNA Directional Library Preparation Kit (MGI, China) prior to standard quality control for sequencing *via* the BGIseq500 platform (BGI, China). The fastq files were preprocessed using fastp <0.23.1>, and gene alignments were performed using the software sTAR <2.7.9a> to Human GRCh38 (hg38); then, gene expression was calculated using HTSeq software. Differential gene analysis was obtained by DESeq2 < v1.4.5>, and the entry criteria for differential genes was (padj < 0.1 and abs(log2FoldChange)>=1).

### Statistical analysis

Statistical analyses were performed with GraphPad Prism 8.0 software using one-way ANOVA with Tukey’s correction for multiple comparisons, paired or unpaired Student’s *t*-tests (two-tailed), and the log-rank (Mantel–Cox) test as appropriate and indicated in each figure. Significant differences were marked on figure legends as *≤0.05, **≤0.01, ***≤0.001, and ****≤0.0001. Two biological replicates at least per experiment, each of which has at least three technical replicates. Experiments with a single biological replicate are *in vivo* experiments.

## Results

### Efficient generation of triple gene–edited universal chimeric antigen receptor T cell with CRISPR/Cas9

**Figure 1:**
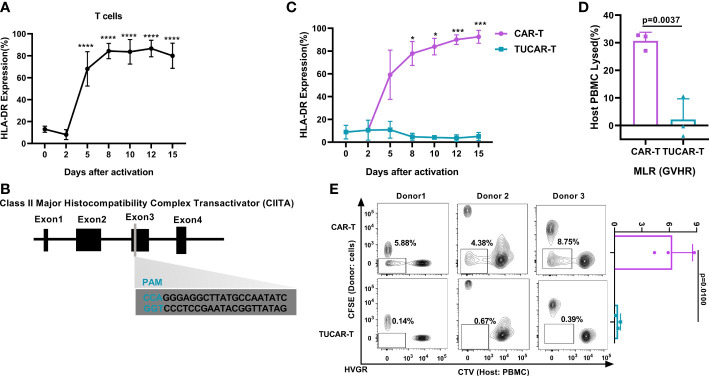
Efficient and specific editing of triplex genes in primary T cells. **(A)** HLA-DR was expressed continuously with the activation of T cells (n = 3). **(B)** Schematic diagram of the designed sgRNA targeting the human CIITA. **(C)** HLA-DR gene expression monitoring in TUCAR-T and unedited CAR-T cells with continued activation (n = 3). **(D)** (n = 3) and **(E)** (n = 3) Alloreactivities between donor TUCAR-T cells and PBMCs from the allogeneic donor were analyzed by the MLR assay. In GvHR, we showed the percentages of host PBMCs that died by rejection lysis **(D)**. In HvGR, the cells in the box represented host PBMCs proliferating from allogeneic CAR-T cell stimulation **(E)**. All data represent the mean ± SD with individual donors. Statistical significance was determined using one-way ANOVA with Dunnett’s correction for multiple comparisons **(A)**, two-way ANOVA with Sidak’s correction for multiple comparisons **(C)**, and two-tailed, paired or unpaired Student’s *t*-test **(D, E)**. Significances of p≤0.05 are indicated by 1 asterisk (*), p≤0.001 are indicated by 3 asterisks (***), p≤0.0001 are indicated by 4 asterisks (****).

Off-the-shelf CAR-T cells using a gene-editing technique to obtain TCR^neg^ and/or HLA-I^neg^ have been extensively reported (13, 14), and relative clinical trials have been conducted (4, 5, 15). As a common gene cluster that mediates acute immune rejection in organ transplantation (16, 17), HLA-II had not drawn sufficient attention in UCAR-T applications. This could be attributed to the low basal expression level of HLA-II in resting T cells. Nonetheless, we have noticed in our previous human clinical studies that HLA-DR was highly presented in autologous CAR-T cells for reinfusing (**Figure S1A**). Further exploration revealed that the expression of HLA-DR molecules increased along with the continued activation of T cells, rising up to 90% on day 9 (**Figure 1A**). Considering this hazard, we aimed to obtain a new UCAR-T cell by eliminating HLA-II, in addition to the elimination of TCR and HLA-I, which was expected to be more resistant to the rejection of the host (**Figures S1B, C**). The CIITA is the master regulator of MHC II expression, which could potentially lead to the accelerated rejection of infused allogeneic T cells (18); CIITA disruption produced a high level of HLA-II deficiency (7). Accordingly, a guide RNA (gRNA) targeting the exon3 of the CIITA gene was designed (**Figure 1B**). Based on our previously reported CD19-targeted CAR (10), we further utilized sgRNA in complex with Cas9 protein (RNP), which was a newly emerging technique with less cellular toxicity for industrial demands after plasmids and viruses. RNP complexes were obtained by incubating sg-TRAC, sg-B2M, and sg-CIITA with Cas9 protein at a molar ratio of 1:1:1:3.

Compared with a continuous high expression of HLA-DR on unedited activated CAR-T cells (**Figure 1C**), over 99% of the CAR-T cells lost CD3, 99% lost HLA-I, and 98% lost HLA-II (**Figures S1D, E**). We also excluded the potential effect of the electroporation stimulus on the expression of HLA-II (**Figure S1F**). The successful elimination of HLA-II on TUCAR-T was further confirmed by continuing the low expression of HLA-II upon T-cell activation (**Figure 1C**). The occurrence of insertions or deletions (indels) in the targeting region of the CIITA gene were established by clonal sequencing (**Figure S1G**). Importantly, there was no predicted off-target events observed in tested TUCAR-T cells (**Table S1**). The mixed lymphatic reaction (MLR) assays were then performed by mixing TUCAR-T donor cells and host PBMCs from allogeneic healthy volunteers, and our data showed that TUCAR-T cells did not induce detectable allogeneic rejection both in GvHR and HvGR compared to unedited CAR-T (**Figures 1D, E**).

### Triple gene–edited universal chimeric antigen receptor T cell has comparable antitumor efficacy with unedited chimeric antigen receptor T cell *in vivo* but exhibited less persistence

**Figure 2:**
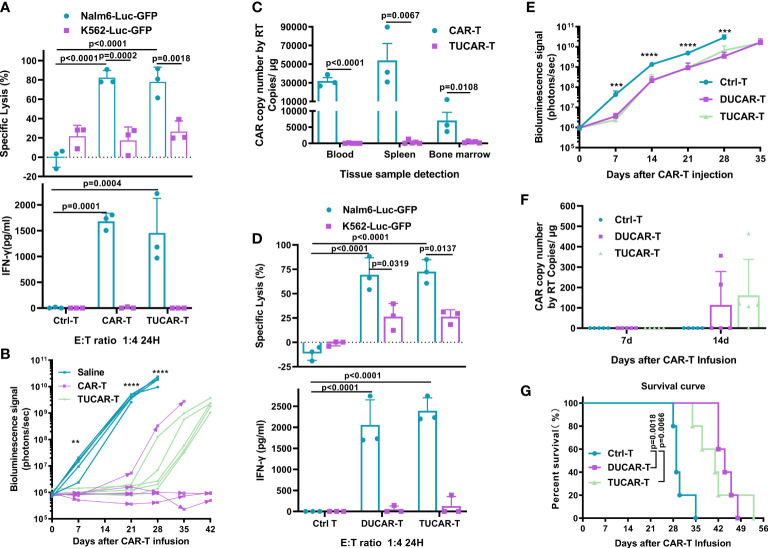
TUCAR-T has comparable antitumor efficacy *in vivo* with unedited CAR-T cells but exhibited less persistency. **(A)** (Top) Cytotoxicity of CAR-T and TUCAR-T cells was assessed by measuring the percentage of tumor cell lysis using the luciferase assay. (Bottom) IFN-γ release was analyzed by ELISA (n=3). **(B)** BLI of mice receiving different treatments (n=5). **(C)** Peripheral blood, spleen, and bone marrow from Nalm6-bearing NOG mice treated with different CAR-T cells were obtained on day 42 after CAR-T infusion for the presence of the copies of the CAR transgene by RT-PCR (BBz) (TUCAR-T: n=5, CAR-T: n=3). **(D)** (Top) Cytotoxicity of DUCAR-T and TUCAR-T cells was assessed by measuring the percentage of tumor cell lysis using the luciferase assay. (Bottom) IFN-γ release was analyzed by ELISA (n=3). **(E)**, BLI from each group of mice (n = 5). **(F)**, Peripheral blood from Nalm6-bearing NOG mice treated were obtained on day 7 and 14 for the presence of copies of the CAR transgene by and RT-PCR (BBz) after CAR-T cell injection (n = 5). **(G)**, Survival curve of mice (n=5). All data represent the mean ± SD. Statistical significance was determined with two-tailed, unpaired Student’s *t*-test **(C)** and one-way ANOVA with Tukey’s correction for multiple comparisons **(A, B, D, E, F)**, or the log-rank (Mantel–Cox) test **(G)**. Significances of p≤0.01 are indicated by 2 asterisks (**), p≤0.001 are indicated by 3 asterisks (***), p≤0.0001 are indicated by 4 asterisks (****).

To test whether CRISPR/Cas9 gene editing would affect the efficacy of CAR-T cells, CD19-specific cytotoxicity and the corresponding interferon-gamma (IFN-γ) secretion of TUCAR-T cells were examined. The results showed that TUCAR-T cells exhibited comparable cellular efficacy in killing CD19^+^ Nalm6-Luc-GFP tumor cells to unedited CAR-T cells *ex vivo* (**Figure 2A**). Both TUCAR-T and unedited CAR-T were effective in controlling tumor growth within 3 weeks of treatment for Nalm6 tumor-bearing mice. However, TUCAR-T failed to keep the effect afterward, while unedited CAR-T worked much better (**Figure 2B**). Consistently, CAR-T cells were undetectable in peripheral blood, the spleen, and bone marrow in the TUCAR-T group but were persistent in the unedited CAR-T group (**Figure 2C**). These results indicated that there was still a certain gap in persistence between TUCAR-T and unedited CAR-T cells.

A previous study had shown that TCR and HLA-I double-edited UCAR-T (DUCAR-T) had comparable antitumor efficacy with unedited CAR-T *in vivo* (14). We thus wondered whether the compromised *in vivo* efficacy of TUCAR-T cells could be due to the knockout of CIITA. To test this conjecture, DUCAR-T cells were produced by the electroporation transduction of the sg-TRAC and sg-B2M RNP mixture. Both TUCAR-T and DUCAR-T showed robust tumor cell lytic capacity and equivalent IFN-γ secretion *ex vivo* (**Figure 2D**). Furthermore, they showed equivalent antitumor capability (**Figure 2E**), similar levels of the CAR copy number in blood on day14 after CAR-T injection (**Figure 2F**), and comparable survival rates in Nalm6 tumor-bearing mice (**Figure 2G**). All these demonstrated that knocking out CIITA in addition to TRAC and B2M did not affect the antitumor ability and persistence of CAR-T cells.

### Introduction of HLA-E into exogenous HLA-E expressing triple gene–edited universal chimeric antigen receptor T cell avoided rejection from host NK cells

**Figure 3:**
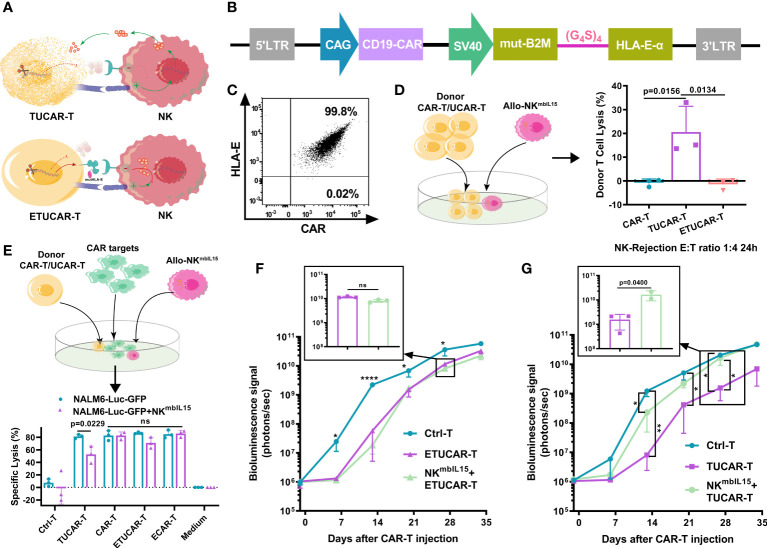
The HLA-E helps UCAR-T cells to escape the lysis induced by NK cells *in vivo* **(A)**, Schematic representation of HLA-I deficient TUCAR-T cells attacked by NK cells. **(B)**, Schematic design of the ECAR constructs. **(C)**, The representative data of HLA-E and CAR coexpressed on T cells by FCM. **(D)** (Left) Schematic of the model setup for UCAR-T cells rejected by NK^mbIL15^. (Right) TUCAR-T cells were lysed by NK^mbIL15^; NK^mbIL15^ and donor UCAR-T cells were mixed at a 1:4 ratio for 24 h (n = 3 ). **(E)** (Top) Schematic diagram of the simulated cell killing *in vivo* with Allo-NK^mbIL15^. (Bottom) Cytotoxicity of ETUCAR-T and TUCAR-T cells was assessed by measuring the percentages of tumor cell lysis using the luciferase assay (n = 3). The tumor cell lysis ratio is 1:4 by the effective cell count; the allo-rejection cell lysis ratio is 1:1 by the total cell count. **(F, G)** BLI from each group of mice (n = 3). All data represent the mean ± SD. Statistical significance was determined with two-tailed, unpaired Student’s *t*-test **(E)** or one-way ANOVA with Tukey’s correction for multiple comparisons **(D, F, G)**. Significances of p≤0.05 are indicated by 1 asterisk (*), p≤0.01 are indicated by 2 asterisks (**), p≤0.0001 are indicated by 4 asterisks (****). Significance of p>0.05 are indicated by nonsignificant (ns).

The recognition of HLA-I by receptors on the surface of NK cells is an important mechanism of immune protection in organisms (8). Therefore, the CD52 monoclonal antibody is commonly adopted for lymphatic clearance prior to the infusion of HLA-I eliminated UCAR-T in clinic to help TUCAR-T cells escape from the rejection of host NK cells, while avoiding lymphatic clearance with anti-CD52 antibodies, which has many adverse effects in clinic. A fusion protein B2M and HLA-E, a non-classical conservative member of HLA-I family, was exogenously constructed to compensate for the elimination of HLA-I (**Figures 3A, B**). Recently, Guo had reported that the introduction of a mutated HLA-E or HLA-G in CAR-T cells along with HLA-I deficiency could help to avoid such rejection (19). However, the study failed to provide *in vivo* evidence to demonstrate its efficacy, and the mutation design of HLA-E was neither uncovered (19). Another report published excellent research in this area but only directly demonstrated *ex vivo* that UCAR-T could resist NK rejection effectively (20). In this study, we introduced mutants at the signal peptide region of wild-type B2M in fusion protein B2M and HLA-E to avoid recognition and cleavage by CRISPR-Cas9 targeting B2M. We confirmed that mutated HLA-E was successfully coexpressed with CAR on the surface of cells (**Figure 3C**).

To verify the protective role played by the expression of HLA-E, we used an armed NK that expresses membrane-bound IL15 (NK^mbIL15^) to enhance the function of NK (**Figures S2A–C**) (21). First, UCAR-T cells were cocultured with NK^mbIL15^. Approximately 20% of TUCAR-T was lysed by NK^mbIL15^, while the ETUCAR-T expression of additional HLA-E was successfully escaped from killing (**Figure 3D**). Furthermore, we assessed the tumor-killing function of ETUCAR-T in the presence of NK^mbIL15^ to emulate the circumstances of CAR-T infused into patients. First, we confirmed that the efficacy of TUCAR-T against Nalm6 tumor cells was significantly attenuated in the presence of NK^mbIL15^. Then, after HLA-E was introduced, the antitumor efficacy of ETUCAR-T remained and performed as well as unedited CAR-T and exogenously introduced HLA-E CAR-T(ECAR-T) (**Figure 3E**). Therefore, the expression of mutated HLA-E indeed endowed UCAR-T with the ability to resist alloimmune rejection mediated by NK. We then performed *in vivo* assessment by the coinfusion of ETUCAR-T or TUCAR-T at a 1:1 ratio with NK^mbIL15^ into Nalm6 tumor-bearing mice. We demonstrated beforehand in the tumor model that NK ^mbIL15^ did not exhibit a specific antitumor activity (**Figure S2D**). Consistent with the *ex vivo* results, the antitumor efficacy of ETUCAR-T-treated mice was maintained (**Figures 3F**, **S2E**), while the antitumor efficacy of TUCAR-T-treated mice was decreased significantly (**Figures 3G**, **S2F**). These findings indicated that exogenously constructing an HLA-E could help UCAR-T cells escape from the cell lysis of host NK and benefit for cell persistence *in vivo*. Our data thus offer an additional possibility for universal CAR clinical applications.

### Multiple infusions of high dose of exogenous HLA-E expressing triple gene–edited universal chimeric antigen receptor T cell could be used as a clinical indication for dosing

**Figure 4:**
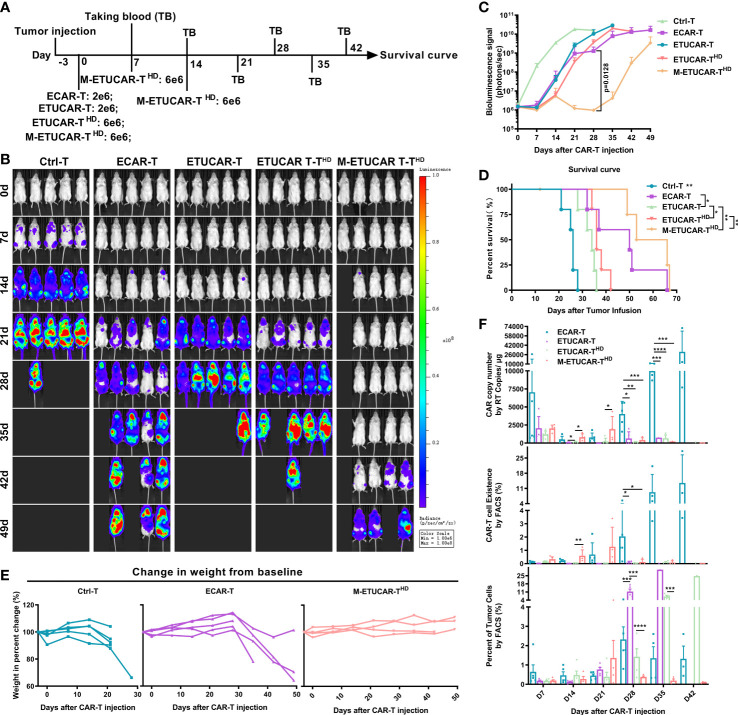
Improve efficacy by increasing the dosage and frequency of CAR-T infusion. **(A)**, Animal experimental timeline. All cells were cryopreserved (off the self). Here, the dose of cryopreserved cells was double the conventional dose (2 × 10^6^). **(B, C)** BLI from each group of mice (n = 5); the high-dose ETUCAR-T^HD^ was three times conventional dosing (2 × 10^6^) reinfusion, the three times conventional dosing for multiple reinfusion group (M-ETUCAR-T^HD^). **(D)**, Survival curve of mice (n=5 per group). **(E)** Weight loss monitoring of mice receiving Ctrl-T (left), ECAR-T (center), or M-ETUCAR-T^HD^ (right) cell treatment (n = 5). **(F)**, Peripheral blood from mice receiving different treatments were obtained every 7 days for the presence of the copies of the CAR transgene by RT-PCR (BBz) (top) and the Fluorescence-activated Cell Sorting (FACS) assay (CD45(+)/CD3(+) CAR-T cells) (center) and the presence of tumor cells (bottom) (n = 5). All data represent the mean ± SD. Statistical significance was determined by one-way ANOVA with Tukey’s correction for multiple comparisons **(C, F)**, or the log-rank (Mantel–Cox) test **(D)**. Parts of the statistical significance in C were not marked.

Given that UCAR-T cells have an inferior clinical efficacy in comparison to unedited autologous CAR-T, we then further tested whether we could overcome this disadvantage by increasing the dosage and frequency of infusions in mice (**Figure 4A**). Furthermore, the UCAR-T transfusion dose was tended as more than three times the autologous CAR-T in clinical trials (1, 22). Nalm6 tumor-bearing mice were established by inoculating Nalm6 and were treated with a single dose of ETUCAR-T or unedited ECAR-T and a single high dose of ETUCAR-T^HD^ or multiple high doses of M-ETUCAR-T^HD^. Thereafter, peripheral blood was collected every 7 days to detect the existence of CAR-T. As we predicted, the increased dose and times of infusion significantly enhanced the antitumor efficacy and prolonged the survival of tumor-bearing mice (**Figures 4B–D**). More importantly, daily observation and weight measurement showed that no accidental death or obvious weight loss was observed in mice treated with a repeated high dose of CAR-T (**Figures 4E**, **S3**). Thus, this indicated that the dosage regimen was safe and effective for treatment. Unfortunately, even though the M-ETUCAR-T^HD^ exhibited better antitumor efficacy, the mice suffered tumor recurrence on day28, approximately 2 weeks after the last treatment (**Figure 4C**). This phenomenon was consistent with the absence of CAR-T cells at this time point (**Figure 4F**). In contrast, the unedited ECAR-T showed higher persistence accompanied by the significant weight loss of mice (**Figures 4E, F**). In conclusion, these results prospectively offered some useful information for the future clinical application of off-the-shelf CAR-T cells. Aiming to advance the clinical use of UCAR-T products and explore the causes and solutions to the industry’s dilemma based on this foundation, the data would serve as an important guideline for clinical trials that need to be done in a short time to facilitate the drug development process in a quicker manner.

### T-cell receptor deficiency in universal chimeric antigen receptor T cell is the primary factor for the inferior efficacy

**Figure 5:**
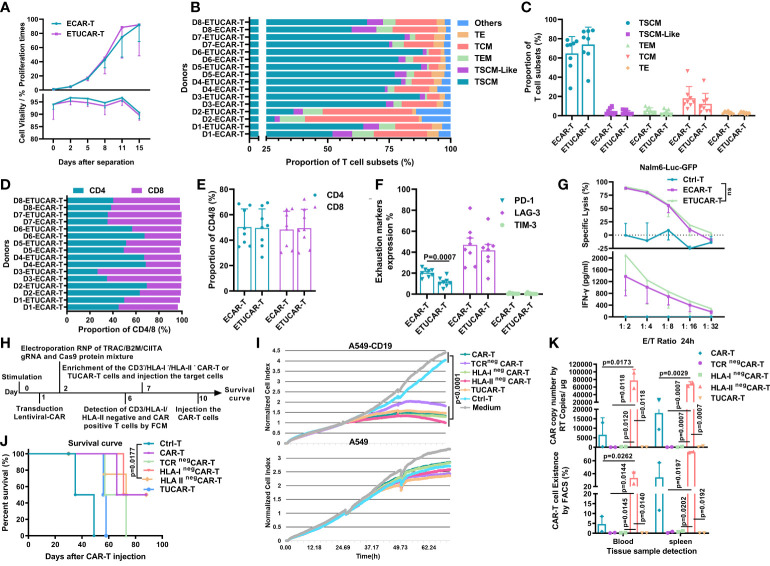
TCR deficiency in UCAR-T cells is the primary factor leading to inferior efficacy compared to unedited CAR-T cells. **(A)**, Comparison of the ETUCAR-T and unedited ECAR-T cell proliferation times (top) and viability (bottom) *ex vivo* (n = 3). **(B, C)**, T-cell subset distributions (n=8). The classification criteria are as shown in **Figure S4**. **(D, E)**, Proportion of CD4/CD8 T cells (n = 8). **(F)**, Cell surface expression of exhaustion markers, programmed cell death protein 1 (PD-1), lymphocyte activation gene-3 (LAG3), and T-cell immunoglobulin and mucin domain-containing protein 3 (TIM-3) (n = 8). **(G)**, Representative data of cell lysis (top) and IFN-γ secretion (bottom) in different E/T ratios (n=3). All data represent the mean ± SD. Statistical significance was determined with two-tailed, unpaired Student’s *t*-test. **(H)**, Flow chart of the generation of UCAR-T cells and the time nodes of other experiments. **(I)**, Cytotoxicity of different UCAR-T cells was assessed by measuring the normalized cell index using RTCA (n = 3). **(J)**, Survival curve of mice (n = 4). **(K)**, Peripheral blood and spleen from mice treated with CAR-T cells was obtained on day 30 for the presence of copies of the CAR transgene by RT-PCR (BBz) (top) and the Fluorescence-activated Cell Sorting (FACS) assay (CD45(+)/CD3(+) CAR-T cells) (bottom). Data from two mice were shown. All data represent the mean ± SD. Statistical significance was determined by two-way ANOVA with Tukey’s correction for multiple comparisons **(A)** or two-tailed, unpaired Student’s *t-*test **(C, E, F)**, log-rank (Mantel–Cox) test **(J)**, or one-way ANOVA with Tukey’s correction for multiple comparisons **(G, I, K)**.

Other research has indicated that the antitumor efficacy of CAR-T cells was correlated with viability, proliferative capacity, T-cell subset distribution, and the CD4/CD8 ratio and could be represented by the expression of exhaustion markers (23–27). Before comparing these indicators, we first excluded the effects of electric shock operation on T cells by a comparative experiment (**Figure S4**). We found that ETUCAR-T was equivalent to unedited ECAR-T in the proliferative capacity and distribution of cell subpopulations or cell exhaustion (**Figures 5A–F**). In addition, representative data showed that they had similar efficacy, which was demonstrated by tumor cell lysis and IFN-γ secretion at different E/T ratios (**Figure 5G**).

With these results, neither the HLA-II deficiency nor the CAR-T subset distribution reflected the key issue, which was responsible for the inferiority of UCAR-T efficacy. To further unravel the crucial factor affecting the efficacy of UCAR-T, we then focused on TCR and HLA-I, the other two genes eliminated in UCAR-T. Despite the fact that the primary function of TCR had been mimicked or replaced by our CAR gene, the endogenous TCR was reported to be involved in many important biological processes (28–32), and HLA-I has also been proven to participate in a diverse range of ways in T cells (31, 32). We thus performed a series of *ex vivo* and *in vivo* comparative studies to explore the differences between the individual or triplex gene-edited CAR-T cells and the unedited CAR-T (**Figure 5H**). We firstly compared the *ex vivo* antitumor capacity of CAR-T utilizing an exogenous construct of CD19^+/-^ A549 by a real-time cell analysis (RTCA) system, which could provide a real-time and informative view of CAR-T killing capacity continuously (33). The data showed that all tested CAR-T exhibited robust and indistinguishable antitumor efficacy *ex vivo* (**Figure 5I**). Next, we further explored their therapeutic efficacy and CAR-T persistence *in vivo*. We found that HLA-I or HLA-II elimination did not affect the antitumor efficacy of CAR-T, and these groups showed the comparable survival rate to the unedited CAR-T (**Figure 5J**). Of note, the deficiency of TCR showed the worst therapeutic effect (**Figure 5J**). In contrast, the CAR-T persistence result revealed a different landscape. The HLA-II^neg^ group showed a markedly high number of CAR-T persistence in blood and spleen after 30 days of treatment, followed by the unedited group, and CAR-T cells were undetectable in either the TCR^neg^ or HLA-I^neg^ group (**Figure 5K**). Collectively, the result suggested that the deficiency of both TCR and HLA-I caused poor CAR-T persistence, but, different from HLA-I, which did not impair the survival rate of treated mice, TCR deficiency was more likely the primary factor leading to the inferior efficacy of UCAR-T.

### Poor efficacy of universal chimeric antigen receptor T cell is associated with a unique transcriptional profile in the absence of T-cell receptor

**Figure 6:**
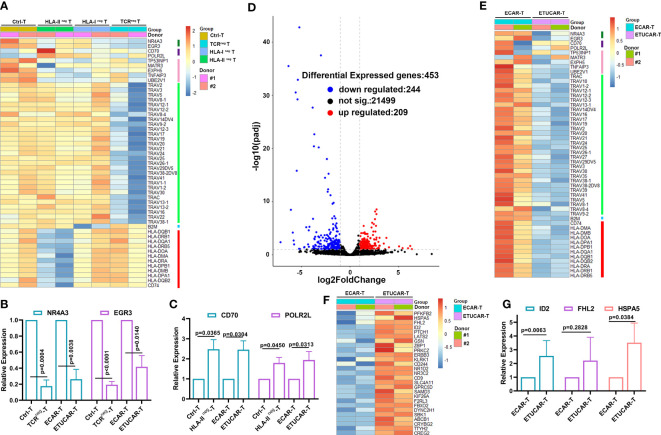
Poor efficacy of ETUCAR-T cells is associated with a unique transcriptional profile. **(A)**, Differential expression gene among Ctrl-T cell transduction with Cas9 protein, TCR^neg^ T, HLA-I^neg^ T, and HLA-II^neg^ T cells was analyzed by RNA sequencing. Heat map of differential expression gene between Ctrl-T and single-gene-deficiency T cells (n = 2). **(B, C, G)**, RT‐PCR results for analyzing the expression of representative differential expression genes in T cells (n = 3). **(D)**, Volcano diagram of differential expression genes of ETUCAR-T cells compared with unedited ECAR-T cells (n = 2). **(E)**, Downregulation expression genes between ECAR-T transduction with Cas9 protein and ETUCAR-T cells were analyzed by RNA sequencing (n = 2). **(F)**, Upregulation expression genes between ECAR-T transduction with Cas9 protein and ETUCAR-T cells were analyzed by RNA sequencing (n = 2). Data represent the mean ± SD. Statistical significance was determined with two-tailed, unpaired Student’s *t*-test. Cells were collected after being activated for 9 days. Differential gene entry criteria were (padj < 0.1 and abs(log2FoldChange)≥ 1).

So far, targeting the TCR and HLA-I is the dominant scheme of research on UCAR-T therapeutic strategies. However, the corresponding change of the transcriptional profiles of gene editing is little known. We thus explored the global transcriptional profiles of TCR, HLA-I, or HLA-II deficiency T cells as well as unedited control T cells from two independent donors to investigate the key genes responding to the poor efficacy of ETUCAR-T. Firstly, the overview of the differential gene expression profile hinted that, unlike TCR^neg^ T, the unedited Ctrl-T and HLA-II^neg^ T were much closer (**Figure 6A**), which implied that HLA-II deficiency had less impact on T cells compared to the other two genes. Furthermore, we verified the dramatic downregulation of gene-editing related genes, including TCR spliceosomes in TCR^neg^ T and B2M in HLA-I^neg^ T, as well as HLA-II isoforms or its invariant peptide chain CD74 in HLA-II^neg^ T, respectively, and all of them in ETUCAR-T. Of note, previous studies have shown that NR4A3 and EGR3 are critical in T-cell survival and differentiation (34–39), but they both showed obvious downregulation in TCR^neg^ T compared to the others (**Figures 6A, B**). These findings may explain the poor *in vivo* efficacy of both TCR^neg^ CAR-T and TUCAR-T, compared to unedited CAR-T (**Figures 5J, K**). Considering the outstanding performance of the *in vivo* persistence of HLA-II^neg^ CAR-T, we analyzed the differences between HLA-II^neg^ T and others. We interestingly found that CD70 and POLR2L were significantly upregulated in the HLA-II^neg^ group (**Figures 6A, C**). CD70 has been known to positively regulate T-cell proliferation (40), whereas the upregulation of POLR2L could also promote T-cell expansion (41). Together, the upregulation of them may facilitate the proliferation and persistence of HLA-II^neg^ CAR-T *in vivo*. Additionally, we noticed that both genes in HLA-I^neg^ were consistent with their TCR^neg^, which might be related to undetectable CAR-T persistence in HLA-I^neg^ (**Figure 6A**).

To explore the comprehensive impact on genes edited in ETUCAR-T, we further analyzed the altered transcriptional profiles compared with CAR-T expressing HLA-E alone. There were 209 upregulated and 244 downregulated genes in ETUCAR-T (**Figures 6D, E**). Moreover, we found a subset of significantly upregulated genes involved in controlling cellular functions, including the negative regulation of cell proliferation such as ID2, LATS2, and PTCH1; the negative regulation of transcription including FHL2, the positive regulation of cell proliferation such as PRKCZ and ERBB3; and the positive regulation of glycolytic processes as PFKFB2 (**Figures 6F, G**). These genes may collectively result in the weakened persistence of ETUCAR-T cells *in vivo*. We also compared the gene panel of ETUCAR-T with TUCAR-T to further to investigate the effects of expressing HLA-E and got a similar transcriptional pattern (**Figure S5**). This suggested that the expression of HLA-E was safe and HLA-E was not the crucial factor for the impaired function of CAR-T. In the summary of these results, the simultaneous editing of all three genes on T cells produced a double-edged result, which reminded us that what we have seen through our experiments was only the tip of the iceberg of the effects of gene editing, and additional information needs to be further explored in-depth. Briefly, these results highlighted the necessity of identifying the potential safety risks of the multiple impacts produced by gene deficiencies when utilizing gene-edited cells as therapeutic transplants in the future.

## Discussion

Allogeneic universal CAR-T therapy has been continuously researched and explored for the benefit of cancer patients who failed to meet the criteria of traditional autologous CAR-T (2). However, the clinical results of universal CAR-T-cell therapy did not reach a parallel level to the autologous CAR-T therapy (4, 5, 15). Currently, the recognized contributors of the struggle for the field have been focusing on poor UCAR-T expansion and survivability *in vivo* (4).

It is known that the risk of HvGR and GvHR in allografts is a key determinant of success, and HLA matching is an important consideration in assessing these risks. Up to now, clinical regimens pay main attention to the elimination of HLA-I, whereas HLA-II was neglected. Herein, we designed a novel universal CAR-T cell called ETUCAR-T, which is designed using CRISPR/Cas9 to eliminate TCR, HLA-I, and HLA-II and incorporates exogenous expression of HLA-E simultaneously. On one hand, ETUCAR-T was more tolerant to host rejection owing to the absence of main MHC molecules. On the other hand, the presentation of HLA-E could assist them to escape the recognition and lysis from allogeneic NK. Multiple infusions of high-dose ETUCAR-T cells in tumor-bearing mice showed no obvious safety issue, suggesting that this regimen was relatively safe and feasible. It was noteworthy that on the research journey of UCAR-T, for the first time, we found that the critical factor for the poor efficacy was the TCR deficiency, and we also found that the HLA-II-knockout improved the persistence of CAR-T *in vivo*. We also revealed the possible key molecules with the RNA-seq analysis of the individual or comprehensive impact of these edited genes.

On the other hand, in this article, no significant difference in antitumor efficacy or T-cell persistence *ex vivo* and *in vivo* were found between reported DUCAR-T and our TUCAR-T, which had the additional elimination of HLA-II (**Figure 2F**). Intriguingly, we found that HLA-II^neg^ CAR-T cells showed superior efficacy and well persistence *in vivo* than TCR^neg^ or HLA-I^neg^ CAR-T (**Figure 5**). Beyond that, the whole transcriptional profile of HLA-II^neg^ T cells is much more similar with unedited Ctrl-T cells (**Figure 6A**). Again, these results supported the necessity and feasibility of HLA-II elimination. It has been suggested that HLA-II expression on T cells could mediate apoptosis through a variety of intracellular signaling pathways (42). Owing to the highly polymorphic characteristics of the HLA-II gene, it was a relatively feasible way to obtain HLA-II-deficient cells by the knockout of CIITA. In addition, previous studies indicated that the DNA methylation of CIITA promoter III in T cells had a great potential for HLA-II deficiency (42), which may bring a new choice for HLA-II elimination. Furthermore, we verified the necessity of HLA-E presence for UCAR-T cells in resisting rejection by allogeneic NK cells both *ex vivo* and *in vivo*. Nonetheless, subsequent clinical trials are essential to validate the role of the exogenous expression of mutant HLA-E in UCAR-T therapy.

With a similar RNP gene-editing scheme, we have successfully produced CD19-targeting UCAR-T cells that could be applied to at least 10 patients by our clinical manufacturing methods. With the rapid development of RNA vaccines in recent years, the large-scale production of RNA has become more sophisticated; thus, this may further support the wide usage of RNP-based gene-editing strategies. Equally important, the data from allogeneic rejection tests and the evaluations of high-dose antitumor infusion demonstrated that simultaneously editing three genes was still safe and feasible. Some researchers in industry now begin to engage in this practice, and our data could provide some support in this area. There is also a trend in the field to conduct gene editing by transducing a single RNA consisting of multiple sgRNAs or siRNAs in a tandem fashion. In addition, the production of RNP complexes manufactured directly by bacteria may become an industry trend (43). However, we should mention that the safety of gene-editing technology remains highly controversial (44). Currently, we have difficulty in claiming whether a large number of gene transcriptional profile changes (**Figure 6**) are caused by gene editing itself or the genes being edited, and whether it is a superimposed effect of both. The two early-starting UCAR-T teams have been urgently suspended by the FDA for safety issues like the occurrence of a clinically lethal event and a report of a chromosomal abnormality in a patient, respectively. These reminded us that more far-reaching impacts caused by gene editing in UCAR-T therapies should be explored in-depth to uncover.

It is well known that endogenous TCR is non-essential for CAR function exertion in CAR-T therapy; nonetheless, in almost all UCAR-T studies reported to date, it has been eliminated by gene editing as a key gene involved in GvHR. Previously published reports barely investigated the irreversible effects of TCR deficiency on T cells; the statements reported to date were in dispute (28, 45). In contrast to Yang (28), as with Stenger (45), our study found that the TCR deficiency contributed to the poor survivability of CAR-T cells, and the lack of effectors would result in the failure of effectively controlling the tumor *in vivo*. It should be noted that the role of TCR deficiency on T-cell persistence might be amplified in a mice model. It had been reported that human TCR could cross-react with MHC molecules in mice, to which T-cell expansion and persistence may benefit (46). We indeed found that some of the mice receiving unedited CAR-T cells developed xenogeneic GvHD at the experiment endpoint. We thus could not exclude the possibility that the inferior persistence of TCR editing CAR-T was a consequence of the elimination of such cross-reaction from mice. Accordingly, better applicable models are needed for the evaluation of treatment efficacy in future studies.

With further exploration, TCR deficiency was found to lead to significant transcriptional profile changes, including the downregulation of NR4A3 and EGR3. NR4A3 is a member of the nuclear receptor subfamily 4, which has been identified as a downstream gene of TCR signaling (34). Previous studies have reported that the NR4A family is essential for maintaining immune homeostasis (36), and NR4A3 regulates Treg cell development (35). EGR3 is a member of the zinc-finger transcription factor in the early growth response gene family that is involved in the development of T cells (37). Previous findings suggested that the EGR3 gene defect in mice accelerated T-cell death as it is involved in the regulation of T-cell antigen recognition (39). Moreover, it has been shown that the lack of EGR2 and EGR3 in lymphocytes led to a fatal autoimmune syndrome and decreased the proliferation of antigen receptor–induced B and T cells (38). For the next investigations, we will systematically validate the functions of these genes to further elucidate the molecular mechanisms involved and reassess the safety risks of gene editing in future studies.

In summary, we have constructed a more effective UCAR-T and provided some new insights into the gene editing of off-the-shelf UCAR-T therapy. Current research on UCAR-T therapy mainly focuses on hematological tumors, such as targeting CD19, CD20, and BCMA while it focuses less on solid tumors, such as targeting NKG2DL and GD2 (2). Actually, UCAR-T would have great advantages in treatment of other diseases that only require short-term effects, such as systemic lupus erythematosus and cardiac disease (47, 48). Joel et al. developed a CAR-T cell for the generation of transient antifibrosis by the lipid nanoparticle (LNP) delivery of CAR’s mRNA *in vivo* and showed that treatment with modified mRNA-targeted LNPs reduced fibrosis and restored cardiac function after injury (48). Comparing the early stage of the *in vivo* manufacture of CAR-T, we believe that UCAR-T could serve the same purpose in the treatment of such diseases. In the flood of UCAR-T against tumors, what we need to do first is to address the poor persistence of UCAR-T, pay attention to the safety risks, and struggle on the development of safe and effective clinical application regimens. We have obtained some hints from RNA-Seq analysis, and with this information, we intend to explore the manifestations of immune rejection–related genes’ absence in the signal pathway of T-cell proliferation and apoptosis. Next, we need to determine the effects of gene editing using CRISPR/Cas9 on cells by comparing the knockout of other genes that are irrelevant to T-cell immune rejection. Additionally, to avoid safety issues that gene editing may bring, we have also focused on non-editing methods for UCAR preparation to acquire inspiration for developing more safe and effective products. For instance, taking advantage of induced pluripotent stem cells, CAR-T can be generated from genomic background–defined clones to overcome the safety issues of gene editing (49). More interestingly, a recent study has successfully prepared universal CAR-T cells by utilizing the mechanism where HIV-1-infected host cells evaded the host immune response by regulating membrane trafficking and achieved the downregulation of MHC-I (50); a combination almost perfectly illustrates the wonders of the life sciences. Up to now, most of the studies in the UCAR industry have been devoted to the development of new products, ignoring the potential pitfalls of gene editing and the genes being edited themselves. In the principle of safety first, we need to pay more attention to mechanism studies, which are indispensable for collaboratively driving the clinical application of the off-the-shelf CAR-T industry.

## Data availability statement

The original contributions presented in the study are publicly available. This data can be found in the NCBI Sequence Read Archive database (Accession: PRJNA905653).

## Ethics statement

The animal study was reviewed and approved by China Council on Animal Care and Chongqing Precision Biotech Co., Ltd. protocol for animal use.

## Author contributions

CQ, JS. and WL contributed to the conception and design, collection and assembly of data, data analysis and interpretation, manuscript writing. XZ, YX performed part of the experiments, discussed the data. JC performed the animal experiments. YL prepared CAR-T cells clinical samples. GW provided suggestions for statistical analysis in data analysis. HZ, ZY, YQ, JH discussed the data and final approval of manuscript. All authors contributed to the article and approved the submitted version.

## Funding

This work was supported by the Major Program of National Natural Science Foundation of China (91959206), Major international (regional) joint research project (82120108019), Key Projects of Ministry of Science and Technology of China (SQ2020YFF0401839).

## Acknowledgments

We would like to gratefully acknowledge all healthy donors who provided peripheral blood for *ex vivo* and *in vivo* studies. We are grateful to Dr. Mengzhu Wang and Dr. Yukang Huang for their helpful suggestions on the manuscript and Mr. Jiabing Ma for the expert advice on the figures. We thank Dr. Huailong Xu for the help with RNA-Seq data analysis.

## Conflict of interest

Chongqing Precision Biotech Co., Ltd. is a biotechnology company focused on research and development of tumor cellular immunotherapy. JS, YX, HZ, JC, ZY, YQ, JH, YL, are full-time employees of Chongqing Precision Biotech Co., Ltd. CQ is the chief scientist of this company.

The remaining authors declare that the research was conducted in the absence of any commercial or financial relationships that could be construed as a potential conflict of interest.

## Publisher’s note

All claims expressed in this article are solely those of the authors and do not necessarily represent those of their affiliated organizations, or those of the publisher, the editors and the reviewers. Any product that may be evaluated in this article, or claim that may be made by its manufacturer, is not guaranteed or endorsed by the publisher.

## Abbreviations

CAR-T, chimeric antigen receptor T cell; UCAR-T, universal chimeric antigen receptor T cell; DUCAR-T, conventional TCR and HLA-I double gene–edited universal chimeric antigen receptor T cell; TUCAR-T, TCR, HLA-I/II triple gene–edited universal chimeric antigen receptor T cell; ETUCAR-T, TCR, HLA-I/II triple gene–edited universal chimeric antigen receptor T cell with an exogenous expression of HLA-E; NK, NK cells isolated from the peripheral blood of a healthy donor; NK^mbIL15^, NK with an exogenous expression of membrane-bound IL15; NR4A3, nuclear receptor subfamily 4 group A member 3; EGR3, early growth response 3; POLR2L, NA polymerase II, I, and III subunit L; GvHD, xenogeneic graft-versus-host disease; GvHR, graft-versus-host reaction; HvGR, host-versus-graft reaction.
