# Supplementary material for: Simultaneous editing of TCR, HLA-I/II and HLA-E resulted in enhanced universal CAR-T resistance to allo-rejection
Source: Front Immunol. 2022 Dec 2;13:1052717. doi: 10.3389/fimmu.2022.1052717 (PMC9757162; doi:10.3389/fimmu.2022.1052717)
Supplement: Supplementary file 1 [file DataSheet_1.pdf]

Supplementary Materials for

Simultaneous editing of TCR, HLA-I/II and HLA-E resulted in enhanced universal CAR-T resistance to allo-rejection

Wuling Li et al.

Corresponding author: Cheng Qian (email: cqian8634@gmail.com) and Junjie Shen (email: junjiesh@gmail.com).

The supplementary figure includes:

Figs. S1 to S5

Other Supplementary Material for this manuscript includes the following:

Supplementary Table 1

Supplementary experimental methods

Supplementary Figures

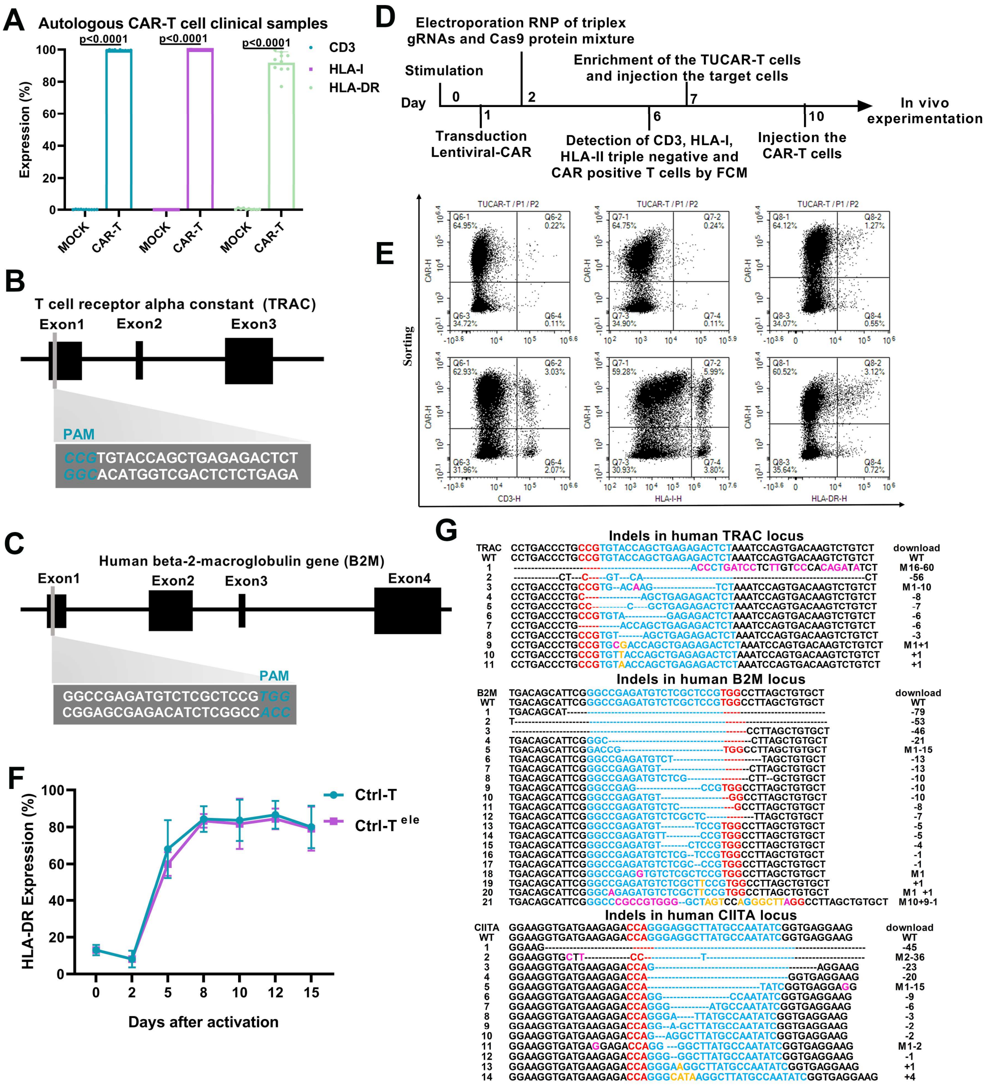

Figure S1| Design and editing efficiency of gene-edited CAR-T cells prepared by RNP delivery systems.

(A), CD3, HLA-I and HLA-DR gene expression in autologous CAR-T cells from different clinical infusion samples (n=10). **(B)** and **(C)**, Schematic diagram of the designed sgRNA targeting the human TRAC **(B)** and B2M **(C)** loci. **(D)**, Flow chart of the generation of UCAR-T cells by RNP delivery. **(E)**, Representative FCM data of CD3/HLA-I/HLA-DR/CAR expression on TUCAR-T. **(F)**, HLA-DR gene expression monitoring in electric-shock T (Ctrl-T^{ele}) cells and unelectric-shock T cells with continuously activated (n=3). (**G),** Indels observed by clonal sequence analysis of PCR amplicons after CRISPR/Cas9 disruption of the TRAC, B2M and CIITA locus. Red indicates the PAM sequence, blue indicates the

target sites, the horizontal bar indicates deletion mutations, pink indicates substitution mutations and yellow indicates insertion mutations. Data were obtained from TUCAR-T cells from 4 donors. Data denote the mean ± s.d. with individual donors. Statistical significance was determined with two-tailed, paired or Student's *t*-test (A) or two-way ANOVA with Sidak's correction for multiple comparisons (F).

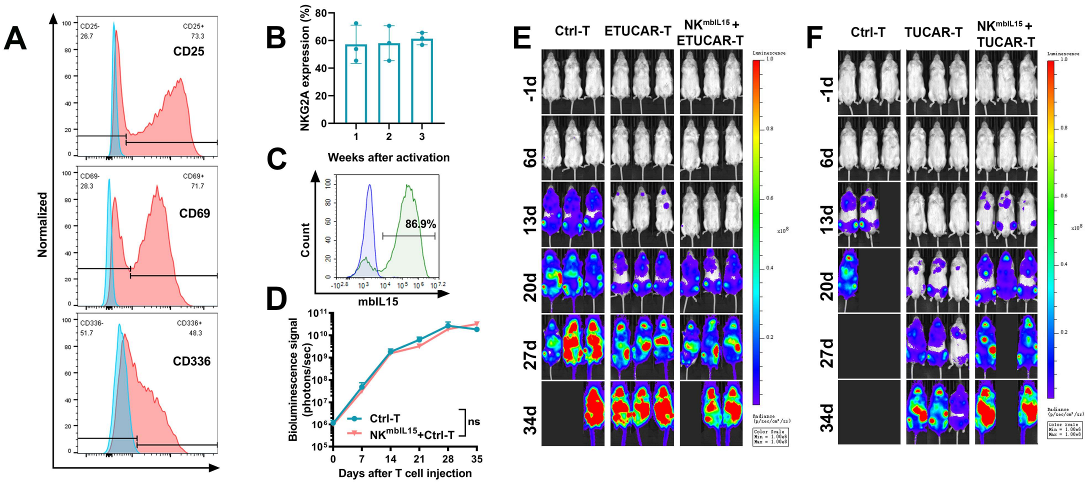

Figure S2| Results of ETUCAR-T cells & mixed NK cells infusion anti-tumor efficacy *in vivo*.

(A), Representative data of the activation status on NK cells on day 9 of activation, blue were the isotype controls. (B), The expression of NKG2A on the activated NK cells (n=3). (C), Representative data of mbIL15 expression in NK, blue was control NK, data represent>3independents with different donors. **(D)**, BLI from each group of mice (n=5). Tumors were established in 8-10 weeks old NOG mice by intravenous injection of 5×10^5 Nalm6 cells on day -3. Beginning on day 0, the NK^{mbIL15} cells (4×10^6 cells were injected 6h before Ctrl-T cells, Ctrl-T cells (4×10^6) were infused with a single injection. Ctrl-T cells were injected as controls. **(E)** and **(F)**, BLI from each group of mice (n=3). Related to Fig.3F and G. Data denote the mean ± s.d. with individual donors. Statistical significance was determined with one-way ANOVA with Tukey's correction for multiple comparisons.

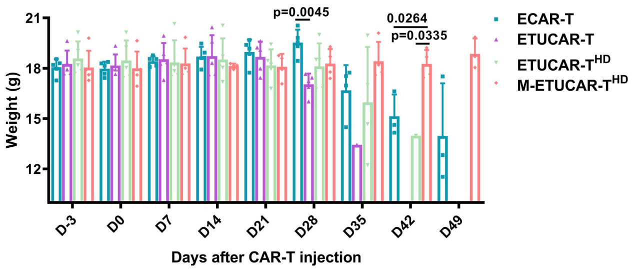

Figure S3| Monitoring of body weight changes in mice.

Data represent the mean ± s.d. Statistical significance was determined by one-way ANOVA with Tukey's correction for multiple comparisons (n=5).

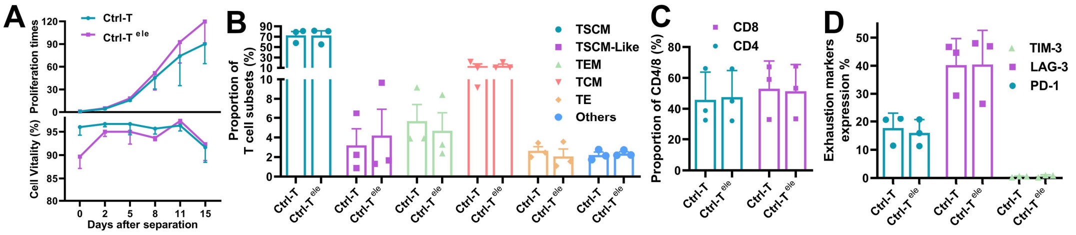

Figure S4| Ctrl-T cells phenotype and viability or proliferation capacity *ex vivo*.

(A), Comparison of the unelectric-shock Ctrl-T and electric shock Ctrl-T^{ele} cells proliferation times (Top) and viability (Bottom) *ex vivo* (n=3). **(B)**, T cell subset distributions (n=3). Classification criteria of T cell subset: TSCM (stem memory T cells) (CD45RA (+) /CD45RO (-)/CD62L (+), TSCM-Like (stem memory T cells-like) (CD45RA (+)/CD45RO (+)/CD62L (+)), TEM (effector memory T cells) (CD45RA (-)/CD45RO (+)/CD62L (-)), TCM (central memory T cells) (CD45RA (-)/CD45RO (+)/CD62L (+)), TE (effector T cells) (CD45RA (+)/CD45RO (-)/CD62L (-)), others (100%-TSCM- TSCM-Like- TEM- TCM- TE). **(C)**, Proportion of CD4/CD8 T cells (n=3). **(D)**, Cell surface expression of exhaustion markers (n=3). All data represent the mean ± s.d. Statistical significance was determined by 2way ANOVA with Tukey's correction for multiple comparisons (A) or two-tailed, unpaired Student's *t*-test (B, C, D).

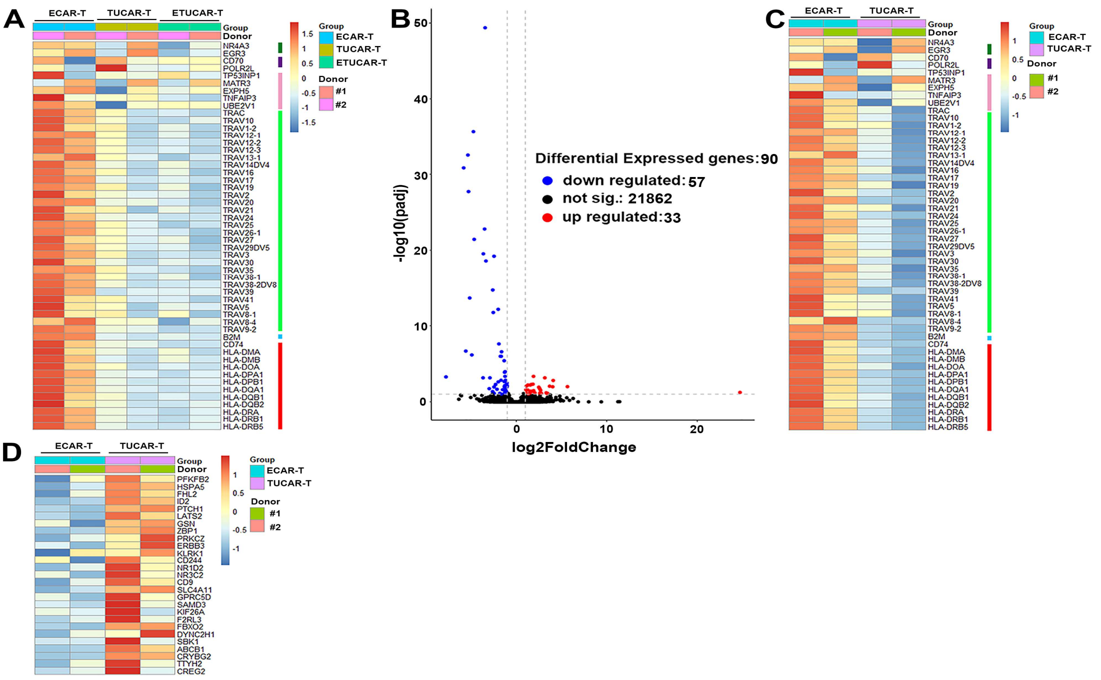

Figure S5| Analysis of RNA-Seq results for TUCAR-T, ETUCAR-T and ECAR-T cells.

(A), Down-regulation expression genes among ECAR-T transduction with Cas9 protein and ETUCAR-T cells or TUCAR-T cells were analyzed by RNA sequencing. **(B)**, Volcano diagram of differential expression genes between ECAR-T and TUCAR-T. **(C)**, Down-regulation expression genes between ECAR-T transduction with Cas9 protein and TUCAR-T cells were analyzed by RNA sequencing. **(D)**, Upregulation expression genes between ECAR-T transduction with Cas9 protein and TUCAR-T cells waere analyzed by RNA sequencing. All results were representative of 2 independent healthy donors. Cells were collected after being activated for 9 days. The threshold of differential expression genes is padj < 0.1 & abs(log2FoldChange) > = 1.

Supplementary Table 1

Table 1 Mutation frequency analysis of the predicted off-target sites.

Target site	gRNA motif (mismatches in red)	PAM	Gene	Off-target analysis (X/Y)
sgTRAC	AGAGTCTCTCAGCTGGTACA	CGG	TRAC	——
Off-target1	TGCCTCCCTCAGCTGGTACA	AGG	HMCN1	0/6
Off-target2	AGTCTCTCTCAGCTGGTGCA	GGG	HPCAL4	0/6
Off-target3	AGAGTCCAGCTGCTGGTACA	TGG	KIR3DL3	0/9
Off-target4	AGAGACTTTCATCTGGCACA	AGG	SLC26A5	0/3
Off-target5	AGAGTATGGCGGCTGGTACA	AAG	3831	0/6
sgB2M	GGCCGAGATGTCTCGCTCCG	TGG	B2M	——
Off-target1	GGCCGGCATCTCTCGCTCCA	CGG	AOC1	0/6
Off-target2	GGCCAAGATGTCTCACACCC	TAG	ZSWIM8	0/6
Off-target3	GGCCCAGAAGTCTGGCTCAG	GAG	ULK4	0/5
sgCIITA	GATATTGGCATAAGCCTCCC	TGG	CIITA	——
Off-target1	CATCTTGGCACAAGCCTCCT	GGG	RAB3D	0/6
Off-target2	AATTTTGGCCTAAGACTCCC	AAG	QSOX1	0/5
Off-target3	GATTTTGGCTGAAGCCGCCC	CAG	27113	0/5

The off-target sites were predicted and aligned with the human genome by Human GRCh38 (hg38).

Supplementary experimental methods

The antibodies used in this study.

From Abcam (England): PE anti-human HLA class I (clone W6/32). From Biolegend (USA): Brilliant Violet 421™ anti-human HLA-DR Antibody (L243), APC anti-human HLA-DR (L243), FITC anti-human CD3 (UCHT1), PE/Cy7 anti-human CD3 (UCHT1), PE anti-human β2-microglobulin (2M2), PE/Cy7 anti-human HLA-E (3D12), PE/Cy7 anti-human CD62L (DREG-56), Brilliant Violet 421™ anti-human CD45RA (HI100), Percp-Cy5.5 anti-human CD45RO (UCHL1), APC anti-human CD279 (PD-1) (EH12.2H7), Brilliant Violet 421™ anti-human CD223 (LAG-3) (11C3C65), PerCP/Cyanine5.5 anti-human CD366 (Tim-3) (F38-2E2), Brilliant Violet 421™ anti-human CD45 (2D1), PE anti-human CD215 (IL-15Rα) (JM7A4), Brilliant Violet 421™ anti-human CD4 (RPA-T4), Brilliant Violet 510TM anti-human CD8 (SK1), PE anti-human CD56 (NCAM) (MEM-188), Biotin anti-human CD3 (UCHT1), Biotin anti-human β2-microglobulin (2M2), Biotin anti-human HLA-DR (L243), APC anti-human CD336 (NKp44) Antibody (P44-8), PE anti-human CD69 Antibody (FN50), FITC anti-human CD25 Antibody (BC96), APC anti-human CD159a (NKG2A) (S19004C). From Jacksonimmune (USA): Alexa Fluor® 647 Streptavidin. From Sinobiological (China): Recombinant Protein L (PE conjugated). From Genscript (USA): Biotin-Protein L.

The primers used in on target and off-target sanger sequencing.

The followed primers for PCR-amplified were used: on-target site of TRAC forward (5′-ACCCTGATCCTCTTGTCCCA-3′) and reverse (5′-AGATTTGTTGCTCCAGGCCA-3′), on-target site of B2M forward (5′-CCTTGTCCTGATTGGCTGGG-3′) and reverse (5′-GACGCTTATCGACGCCCT AA-3′), on-target site of CIITA forward (5′-CACCAGCCCTCTTTCCAGA-3′) and reverse (5′-CCCCTTGCAATGATTTCTGT-3′). Off-target site 1 of TRAC forward (5′-GGCCAGGTCTTTCCCC TAAA-3′) and reverse (5′-TGATTCTCTGAGTCACTTGTGT-3′),off-target site 2 of TRAC forward (5′-CAGGAGCACTGTGGTAGAGC-3′) and reverse (5′-TGCAATTCACAGA GTTGTTGC-3′), off-target site 3 of TRAC forward (5′-CAAATGCTGAGCGCAGATCC-3′) and reverse (5′-TGTGGGCTAAGC AAGTGTGT-3′), off-target site 4 of TRAC forward (5′-TGCTACCCCTAATTATCACTGTCC-3′) and reverse (5′-CTCACCACTGCATCCTGCTA-3′), off-target site 5 of TRAC forward (5′-TGCATGC TGAAGAAAGAGAAGAATG-3′) and reverse (5′-TTCGGGAAAAACTGGGACT-3′), off-target site 1 of B2M forward (5′-AAAATTCCATGGCCCTAACCTGA-3′) and reverse (5′-GGTTGCTTAGGTCT

GAAAACACC-3′), off-target site 2 of B2M forward (5′-CCTACAGAACCTCAAGCGCA-3′) and reverse (5′-AATACCAGAGGCCACCCAAC-3′), off-target site 3 of B2M forward (5′-TTGTGTCTGCCCGATGGAGT-3′) and reverse (5′-TTCAACTTCAGTAATGCCCCAAAA-3′), off-target site 1 of CIITA forward (5′-CTCCGAGTGACCGAGCTGA-3′) and reverse (5′-TCCGCGTATCGGAACAGGA-3′),off-target site 2 of CIITA forward (5′-GTCTAGCCACAACAGGGTCAATG-3′) and reverse (5′-GCTGATGATACTGGCATTGGTTA-3′), off-target site 3 of CIITA forward (5′-AGCCAAACGTGACCACTAGC-3′) and reverse (5′-ATCTACAGCAGCGCATATACAGT-3′).
